# INNOVATIVE COLLABORATION: DEVELOPING DEMENTIA TRAINING WITH STUDENTS, SCHOLARS, AND PRACTITIONERS

**DOI:** 10.1093/geroni/igae098.2979

**Published:** 2024-12-31

**Authors:** John Shean, Eva Jackson, Tara Redd, Madiha Shakir, Shelby Roberts

**Affiliations:** Alzheimer’s Association, Chicago, Illinois, United States; Alzheimer’s Association, Chicago, Illinois, United States; Emory University, Atlanta, Georgia, United States; Full Tilt Ahead, Arlington, Virginia, United States; Alzheimer’s Association, Chicago, Illinois, United States

## Abstract

The growing prevalence of Alzheimer’s and other dementias and the urgent need for public health awareness necessitates innovative, interactive, and accessible dementia training for students entering the workforce. The curriculum, A Public Health Approach to Dementia, is a new tool to meet this need. Through partnership between the Alzheimer’s Association, CDC, and Emory Centers, four new modules were launched in 2023 and 2024. These free, asynchronous, online learning modules support the training needs of the current and future public health workforce. Throughout the development, the team relied on students, researchers, professors and practitioners to ensure the modules provide accessible, comprehensive knowledge on the public health approach to dementia. The curriculum includes courses on the public health impact of dementia, health equity and dementia, and public health and dementia caregiving. Data from the first two modules released in 2023, show that they are impactful. Health Equity in Dementia — Using a Public Health Lens to Advance Health Equity in Alzheimer’s and Other Dementia was released in August and has over 520 people enrolled. Public Health and Dementia Caregiving was released in December and has over 200 people enrolled. Both modules had over 900 registrants for each launch webinar. Across both modules, most learners reported an increase in knowledge after taking the course (89% and 92%) and reported that it will be useful in their work or studies (87% and 90%). This curriculum demonstrates the strengths of partnering across backgrounds and expertise to support innovative learning opportunities for students entering the workforce.

